# East Texas Medical Association

**Published:** 1887-07

**Authors:** J. E. Mayfield

**Affiliations:** Sec. E. T. M. A.; Nacogdoches


### SOCIETY NOTES.

### EAST TEXAS MEDICAL ASSOCIATION.

The cause of medical organization is sufficient apology for this dry communication. The last meeting of the E. T. M. A. was held at Lufkin, July 5th and 6th. Several interesting papers were read and discussed, besides reports of cases and other miscellaneous subjects. The leading aim and feature of this Association is the consideration of medical subjects, spiced with social, professional intercourse, avoiding, as nearly as practicable, expenses and incidental business troubles. Members and others who attend are invariably pleased and edified. The subjects for next meeting, which will be at Alto, Tuesday, October 4, 1887, at 7 p. m., are as follows: Abortion, paper by Dr. Mayfield, discussion opened by Dr. Barham. Phthisis Pulmonalis, paper by Dr. McCord, discussion opened by Dr. Denman. Menstrual Disorders, paper by Dr. Frazer, discussion opened by Dr. Coon. Diarrhœas of children, paper by Dr. Evans, discussion opened by Dr. G. M. Abney. Uterine Displacements, paper by Dr. J. A. Abney, discussion opened by Dr. Treadwell.

A meeting will probably be held at Jacksonville, first Tuesday in January, and a large attendance is then expected. The East Line Medical Association will be consulted as to consolidating or meeting with us. We require no admission fees or dues, and inflict no pecuniary penalties. No limit as to territory of membership, only qualification, *regular*. Attendance free to all. We have fifty-two enrolled members, and want many more.

J. E. MAYFIELD,
NACOGDOCHES, July 12, 1887. Sec. E. T. M. A.

